# Uncommon erythema multiforme in small children: experience of a single Romanian pediatric unit: Two case reports

**DOI:** 10.1097/MD.0000000000017895

**Published:** 2019-11-15

**Authors:** Adriana Mocanu, Anca Ivanov, Mirabela Alecsa, Vasile Valeriu Lupu, Ancuta Lupu, Iuliana Magdalena Starcea, Oana Tatiana Miron, Cristina Gavrilovici, Ingrith Crenguta Miron

**Affiliations:** a Department of Pediatrics, University of Medicine and Pharmacy “Grigore T. Popa”; b IVth Pediatric Department; c Vth Pediatric Department, Sf. Maria Emergency Hospital for Children, Iasi, Romania.

**Keywords:** children, erythema multiforme, immune-mediated disease

## Abstract

**Rationale:**

Erythema multiforme (EM) is an immune-mediated disease with mucocutaneous localization and plurietiologic determinism. The term “multiforme” refers to the variety of aspects that the lesions can take from patient to patient and during evolution in a single patient.

**Patient concerns:**

We have selected 2 cases of small children diagnosed with different etiology of EM to illustrate the importance of a correct and fast diagnosis. Case 1 involves a 2-year-old girl from a rural area who presented with fever and pruritic erythematous papular eruption. The onset of the symptoms was 3 days before presentation with fever and ulcerative lesions on the oral and labial mucosa, followed by the appearance of erythematous macular lesions, with progressive confluence to intense pruritic patches. The 2nd involves a 2-year-old boy with fever, loss of appetite, productive cough, and petechiae. He had corticosensible immune thrombocytopenia from the age of 6 months, with many recurrences. The patient received treatment with ampicillin/sulbactam and symptomatics for an erythemato-pultaceous angina. During the 2nd day of treatment the patient developed an erythematous macular eruption on the face, scalp, trunk, and limbs, with bullae formation.

**Diagnoses:**

The 1st patient was diagnosed based on biologic findings: positive inflammatory syndrome, elevated level of anti-*Mycoplasma pneumoniae* immunoglobulin M antibodies and immunoglobulin E. Histopathologic examination described papillary dermal edema, inflammatory infiltrate, and lymphocyte exocytosis. In the 2nd case, the hemoleucogram identified 12,000/mm^3^ platelets and the medulogram aspect was normal. Serology for Epstein–Barr virus was negative. The diagnosis was EM secondary to *M pneumoniae* infection in case 1 and secondary to administration of ampicillin/sulbactam in case 2.

**Interventions:**

In both cases, etiopathogenic treatment consisting of steroidal antiinflammatory drugs, antihistamines was administered. Because of specific etiology, the 1st case received antibiotics.

**Outcomes:**

The evolution was favorable in 10 to 14 days; the patients were discharged after etiopathogenic treatment consisting of steroidal antiinflammatory drugs, antihistamines, and/or antibiotics.

**Lessons:**

Performing a detailed clinical examination, medical history of drug use, infection or general diseases can establish a good diagnosis of EM. Histopathologic examination can help. The treatment is etiologic, pathogenic, and symptomatic. EM usually has a self-limited evolution.

## 1 Introduction

Erythema multiforme (EM) is an immune-mediated disease with mucocutaneous localization and plurietiologic determinism. It is characterized by the appearance of maculopapular lesions, sometimes vesicles and bullae, with a target-like aspect, self-limited evolution, and little chance of recurrence.^[1]^ The term “multiforme” refers to the variety of aspects that the lesions can take from patient to patient and during evolution in a single patient. The annual incidence of EM in the pediatric population is considered to be approximately 1%, although there are no exact data. Young adults aged 20 to 40 years are most commonly affected by the disease, with a slight male predominance. EM can also be observed in elderly adults and children.^[1]^ We report 2 cases of EM observed in young children. The 1st case was a result of infection with *Mycoplasma pneumoniae*. However, in the 2nd case, it was impossible to establish the diagnosis considering the etiology, but the clinical and biologic evolution led to the establishment of diagnosis.

## 2 Methods

### 2.1 Case 1

**Figure 1:**
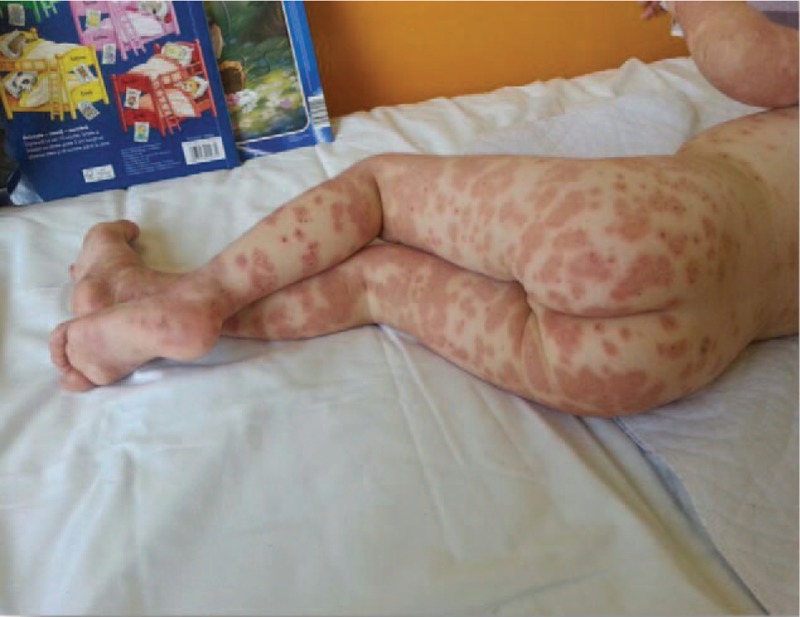
Erythematous skin lesions on inferior limbs.

**Figure 2:**
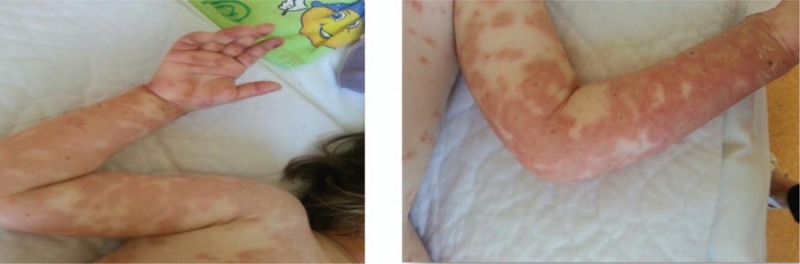
Erythematous skin lesions on upper limbs and buttocks.

**Figure 3:**
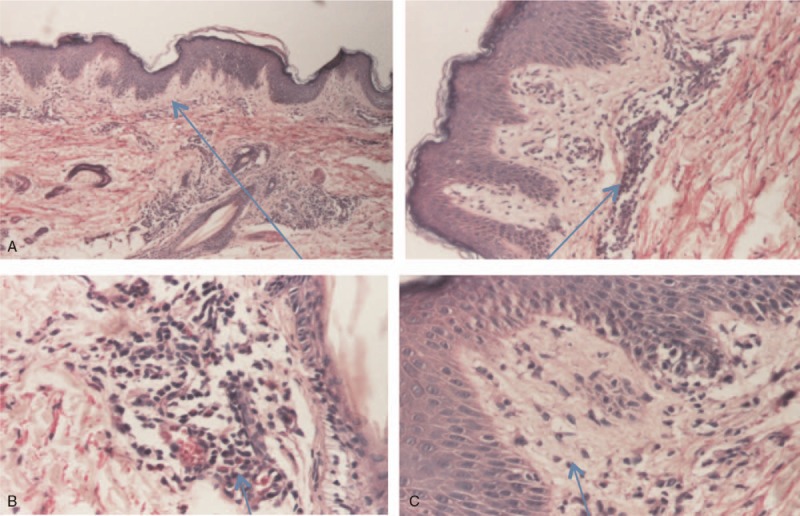
(A) Papillary edema and inflammatory infiltrate. (B) Inflammatory infiltrate (eosinophils and polynuclears). (C) Exocytosis.

**Figure 4:**
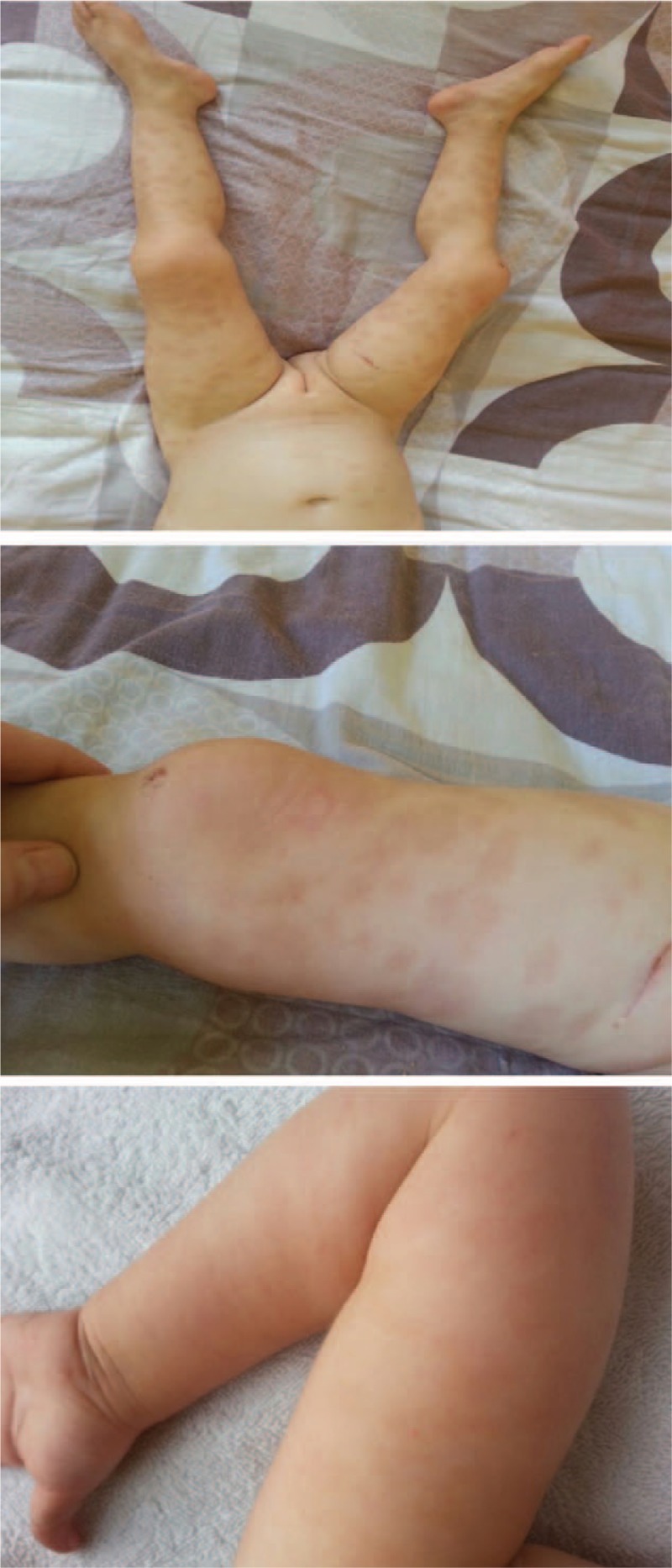
Healing lesions on lower limbs.

A 2-year-old girl from a rural area presented with fever and a pruritic erythematous papular eruption with a tendency to confluence. Symptom onset occurred 3 days prior to presentation. It consisted of fever and ulcerative lesions on the oral and labial mucosa followed by the appearance of erythematous macular cutaneous lesions, with progressive confluence to intense pruritic patches. The patient experienced an episode of upper respiratory tract infection 2 weeks earlier. Clinical examination performed at the time of presentation revealed impaired general status, absence of fever, low appetite, normal breathing and heart rate, soft abdomen with no point of tenderness with pain production, normal passage of feces, and physiologic micturition. Mucous membrane examination revealed ulcerative lesions (oral and labial mucosa) and erythematous maculopapular skin lesions, some of them with target aspect, symmetrical distribution on the extensor surfaces of the extremities, and a tendency to confluence (Figs. 1 and 2). As symptoms evolved, they spread to the abdomen and buttocks. Biologic findings revealed a positive inflammatory syndrome and increased level of anti-*M pneumoniae* immunoglobulin M and immunoglobulin E antibodies. Histopathologic examination revealed papillary dermal edema, inflammatory infiltrate, and lymphocyte exocytosis (Fig. 3 A–C). Examination specific for infectious and dermatologic diseases raised the suspicion of EM. Evaluating the clinical aspect of the lesions, dermatologic and infectious disease examinations, positive serology for *M pneumoniae*, and positive histologic examination, we established the diagnosis of EM. The patient received an etiopathogenic treatment consisting of steroidal antiinflammatory drugs (initially intravenous 8–10 mg/kg/d of hydrocortisone hemisuccinate followed by 0.3 mg/kg/d of dexamethasone), antihistamines (2.5 mL/d of desloratadine), antibiotics (15 mg/kg/d of clarithromycin), and adjuvant therapy with esomeprazole and calcium and topic application of water paste and emollient cream. Under the above treatment, patient's clinical outcome was favorable in 10 days, and the patient was discharged with a good general status and healing lesions (Fig. 4).

### 2.2 Case 2

**Figure 5:**
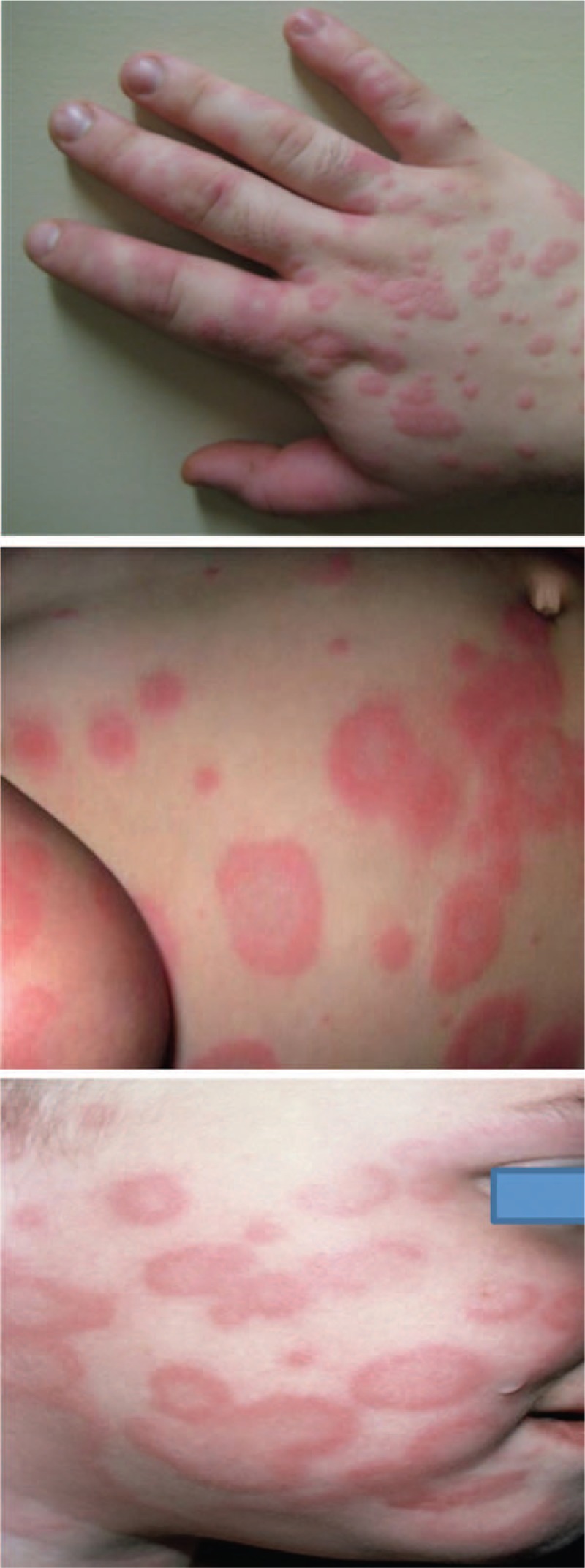
Hand, trunk, and face lesions.

A 2-year-old boy presented with fever, loss of appetite, productive cough, and petechiae. According to the patient's previous medical history, he was diagnosed with immune thrombocytopenia when he was 6 months old, with recurrences, and was treated with 2.5 mg/d of prednisone in an alternate-day therapy. Laboratory findings were as follows: platelet count of 17,000/mm^3^ and negative serology for Epstein–Barr virus. Clinical examination performed at the time of presentation identified an erythemato-pultaceous angina. The patient received ampicillin/sulbactam and symptomatic treatment. During the 2nd day of treatment, the patient developed an erythematous macular eruption on the face, scalp, trunk, and limbs, with vesicles and bullae formation (Fig. 5). It was associated with hypertrophic gingivitis lesions with friable, bleeding mucosa. When laboratory diagnostic testing was repeated, the complete blood count revealed 2810/mm^3^ white blood cells and 12,000/mm^3^ platelet count, but the medulogram was normal. Antibiotic therapy was stopped, and general treatment was initiated with dexamethasone, cetirizine hydrochloride, calcium, vitamin C, and topical steroids with hydrocortisone acetate/fusidic acid cream and dexamethasone/glycerine. The clinical outcome was favorable in 14 days, with healing mucosal and cutaneous lesions. The patient was discharged with 386,000/mm^3^ platelet count. In this case report, we included the administration of ampicillin/sulbactam.

## 3 Discussion

The pathophysiology of EM is not well elucidated, but there is an existing evidence stating that EM is associated with the following several factors: infections, medications, malignancies, autoimmune diseases, immunizations, radiation, sarcoidosis, and menstruation.

A comprehensive search of all PubMed case reports regarding EM in young children revealed 35 articles. Simultaneously, a search on Web of Science showed 9 articles on this topic. Using PRISMA flow diagram for EM in young children removed duplicates and screened 38 full-text articles assessed for eligibility. A total of 18 articles were excluded, and 20 studies were included in the analysis.

The etiology in 90% of cases indicated that the most common precipitating factor is infections, generally herpes simplex virus (HSV) infection. The 1st presented case report clearly revealed that EM was significantly associated with *M pneumoniae* infection due to the presence of the antibody. Since HSV is the most frequent agent related to EM's etiology, there is no surprise that most of the information regarding its pathophysiology comes from the study of EM associated with HSV infection. Finding HSV deoxyribonucleic acid (DNA) in skin biopsy specimens of patients with EM supports the idea of a T-cell-mediated cytolytic reaction directed against the viral antigens present in keratinocytes responsible for the development of EM.^[3]^ Despite the high incidence of HSV infection, not all individuals infected with HSV develop EM. According to literature data, the strongest correspondence with the allele between HLA types A33, B35, B62 (B15), DR4, DQB1∗0301, DQ3, and DR53 were identified among patients with herpes-associated EM.^[6]^ In our current practice, we identified medications responsible for <10% of EM cases. The most common drugs are nonsteroidal inflammatory drugs, sulfonamides, antiepileptics, and other antibiotics.^[1,5]^ In the 2nd case report, the etiology was associated with ampicillin/sulbactam treatment. Cases that describe the association between EM and aminopenicillin use are not found in the literature.

Regarding clinical manifestation, we can classify EM into either a minor, less severe form, affecting 80% of cases, or a major form. The minor EM is described only by skin lesions: big erythematous papule (Ø = 0.5–1.5 cm) with a 72-hour evolution to the classical “target” lesions found on the extremities, while the major form of EM affects both the skin and mucous membranes. “Target” lesions or “bulls-eye” are the characteristic aspects of the cutaneous lesions, but these may not always be present, or sometimes can have an atypical aspect. Usually, the lesions appear symmetrically on the extensor surfaces of the distal extremities and progress proximally to the abdomen and back. It may be generalized and can affect the palms, neck, and face. Mucosal lesions often appear together with skin lesions in major EM and heal with no sequelae, such as in the 2nd case report presented. The buccal mucosal lesions rarely extend to the pharynx and upper respiratory tract.^[7]^ Patients with *M pneumoniae*-related EM may experience respiratory symptoms.^[4,8]^ The course of the disease is usually self-limited with a resolution of lesions within approximately 2 weeks. Rarely, ocular mucosa involvement can result in keratitis, conjunctival scarring, or even visual impairment.

Histopathology results of skin biopsy are useful in establishing the diagnosis by excluding other similar diseases. Pathologic findings in EM typically include basal cell vacuolar degeneration, scattered necrotic keratinocytes, and lymphocyte exocytosis.^[1,9]^ Skin biopsy results vary depending on the clinical morphology and the duration of the lesions’ existence and the area of the lesion from which the specimen is obtained: the central region of the lesion consists of subepidermal separation with necrotic keratinocytes, while the peripheral specimens show predominantly dermal changes, such as papillary dermal edema, vascular dilation, and a perivascular mononuclear cell infiltrate. In the 1st case reported, a typical aspect was found with inflammatory infiltrate and lymphocyte exocytosis.

The diagnosis of EM is usually based on anamnesis and physical examination. Patients with typical lesions and a preceding or coexisting infection or drug use are easily diagnosed with EM. Skin biopsy results and laboratory findings are not specific for EM and are only required for differential diagnosis. Since HSV infection seems to be associated with EM in a majority of cases, this etiology must be taken into consideration when evaluating every new patient. When there are lesions with possible active HSV infection present, the presence of the virus can be confirmed using direct fluorescent antibody, viral culture, Tzanck smear, or polymerase chain reaction (PCR) studies. Testing for HSV is also recommended in recurrent EM. Serologic tests for *M pneumoniae* must be performed in patients with respiratory symptoms to identify the etiology of the eruption, such as in the 1st case report. Even some cases classified as idiopathic EM are considered to be related to subclinical HSV infection, with PCR-HSV DNA being found in lesional skin biopsy specimens.^[3,8,10]^ In distinguishing the episodes of recurrence, we recommend PCR tests instead of serologic test.^[3,8,10]^ Persistent EM belongs to the spectrum of EM, characterized by the continuous appearance of typical and atypical cutaneous and/or mucosal lesions. These are associated with hypocomplementemia and circulating immune complexes, specifically after viral infections, particularly HSV and Epstein–Barr virus, and inflammatory bowel disease and malignancy.

The evolution of the disease without an appropriate treatment can continue for more than 1 year.^[11]^ Literature data identify an average of 6 episodes of EM per year, with a mean duration of 6 to 10 years.^[10]^ However, there are only few typical lesions, or the lesions can have an atypical aspect; hence, a complex differential diagnosis is required.^[12,13]^

The 1st disease often discussed as the differential diagnosis is Stevens–Johnson syndrome (SJS).^[2]^ Although the aspect of the lesions at the debut is similar in both entities, SJS tends to affect the mucous membranes in more than 90% of cases, is characterized by extensive necrosis and detachment of the epidermis, and is usually accompanied with systemic symptoms, and its appearance is often due to drug administration.^[5]^ SJS has rarely been reported in patients on azithromycin therapy.^[13]^ Fixed drug eruption can typically present well-demarcated, round, dusky red to brown/black macules. These can evolve into edematous plaques with or without vesiculation or blistering. It seems to develop everywhere on the body. The acute eruption usually appears 30 minutes to 8 hours after drug administration, and they heal spontaneously after drug cessation. When re-exposed to the drug, the patient develops lesions in the same location and some new sites.^[5]^ A complete history of specific drug ingestion is required for the establishment of the diagnosis.

The therapeutic approach must be based on EM's severity. Usually, EM has a self-limited evolution, and only a few patients’ experience recurrences. Management of EM involves determining the etiology, where possible, and subsequently treating the infection or discontinuing the causal drug.^[11,14]^ The 1st patient, due to clear etiology, received an etiopathogenic treatment consisting of steroidal antiinflammatory drugs, antihistamines, and specific antibiotics. The literature contains data for specific treatment only on HSV-related EM. Suppression of HSV can prevent EM's occurrence, although the antiviral therapy loses its effect once the eruption occurs. However, antiviral therapy must be taken into consideration as a prophylaxis for HSV-associated EM recurrence.^[4]^ Mild cases of EM do not require treatment. Oral antihistamines and topical steroids may be used to provide symptomatic relief.^[11]^ Both of our cases received this kind of treatment. In cases with extensive involvement of the oral mucosa, severe pain, and inability to ingest foods or liquids, initiating systemic glucocorticoid oral therapy is significantly recommended. Painful oral erosions have a good response to topical corticosteroid gel, mouthwashes (mixture of lidocaine, diphenhydramine), and antacids. Patients with debilitating lesions may need to be hospitalized for nutrition and pain control.^[7]^ Ocular mucosa involvement requires an ophthalmologist's assessment for the appropriate management of lesions and prevention of long-term sequelae, usually with topical ophthalmic preparations. As mentioned previously, recurrent HSV-associated EM and idiopathic recurrent EM can benefit from antiviral therapy used as a prophylaxis. Current practice recommends antiviral drugs in patients with more than 5 recurrences per year. Cases with no response to antiviral therapy may benefit from immunosuppressive or immunomodulatory therapies.^[10,18]^ Alternative treatments for EM include dapsone, antimalarials, azathioprine, cimetidine, and thalidomide.^[15–18]^

## 4 Conclusion

The EM is a disease with plurietiologic determinism. Since there are no specific protocols in the establishment of EM diagnosis, performing a detailed clinical examination and a medical history of drug use and determining infection in general diseases are significantly required. Histopathologic examination can help in establishing EM diagnosis. The treatment is etiologic, pathogenic, and symptomatic. EM usually has a self-limited evolution. The major form heals within 3 weeks with hypo- or hyperpigmentation of the affected areas. Pediatricians consider various clinical features, different evolution modalities, and response to treatment for EM, specifically in young children, as the significant challenges when establishing EM diagnosis.

## Acknowledgment

The authors thank Editage (www.editage.com) for English language editing.

## Author contributions

**Conceptualization:** Adriana Mocanu.

**Data curation:** Anca Ivanov, Oana Tatiana Miron.

**Formal analysis:** Vasile Valeriu Lupu, Oana Tatiana Miron.

**Funding acquisition:** Ancuta Lupu, Oana Tatiana Miron.

**Investigation:** Adriana Mocanu, Mirabela Alecsa, Vasile Valeriu Lupu, Ancuta Lupu, Oana Tatiana Miron.

**Methodology:** Ancuta Lupu, Iuliana Magdalena Starcea.

**Project administration:** Adriana Mocanu, Vasile Valeriu Lupu, Iuliana Magdalena Starcea.

**Resources:** Anca Ivanov, Mirabela Alecsa, Vasile Valeriu Lupu, Ancuta Lupu.

**Software:** Anca Ivanov, Mirabela Alecsa.

**Supervision:** Ancuta Lupu, Iuliana Magdalena Starcea, Cristina Gavrilovici, Ingrith Crenguta Miron.

**Validation:** Ancuta Lupu.

**Visualization:** Anca Ivanov, Mirabela Alecsa.

**Writing – original draft:** Adriana Mocanu, Iuliana Magdalena Starcea, Cristina Gavrilovici.

**Writing – review & editing:** Vasile Valeriu Lupu, Cristina Gavrilovici, Ingrith Crenguta Miron.

## References

[1] Stoopler ET Huston AM Chmieliauskaite M Erythema multiforme. J Emerg Med 2015;21:1–2. 10.1016/j.jemermed.2015.06.018 26281815

[2] Lucchese A From HSV infection to erythema multiforme through autoimmune cross reactivity. Autoimmun Rev 2018;17:576–81. 29635075 10.1016/j.autrev.2017.12.009

[3] Langley A Anooshiravani N Kwan S Erythema multiforme in children and *Mycoplasma pneumoniae* aetiology. J Cutan Med Surg 2016;20:453–7. 26976263 10.1177/1203475416639018

[4] Sousa-Pinto B Araujo L Freitas A Stevens–Johnson syndrome/toxic epidermal necrolysis and erythema multiforme drug-related hospitalisations in a national administrative database. Clin Transl Allergy 2018;8:2. 29387340 10.1186/s13601-017-0188-1 PMC5776772

[5] Khalil I Lepage V Douay C HLA DQB1∗0301 allele is involved in the susceptibility to erythema multiforme. J Invest Dermatol 1991;97:697–700. 1940441 10.1111/1523-1747.ep12484029

[6] Ayangco L Rogers RS 3rd Oral manifestations of erythema multiforme. Dermatol Clin 2003;21:195–205. 12622281 10.1016/s0733-8635(02)00062-1

[7] Amode R Ingen-Housz-Oro S Ortonne N *Mycoplasma pneumoniae*-related erythema multiforme: clinical and histological features. A single center series of 33 cases compared to 100 cases induced by other causes. J Am Acad Dermatol 2018;79:110–7. 29559400 10.1016/j.jaad.2018.03.013

[8] Huff JC Weston WL Recurrent erythema multiforme. Medicine 1989;68:133–40. 2654536 10.1097/00005792-198905000-00001

[9] Wetter DA Davis MD Recurrent erythema multiforme: clinical characteristics, etiologic associations, and treatment in a series of 48 patients at Mayo Clinic, 2000 to 2007. J Am Acad Dermatol 2010;62:45–53. 19665257 10.1016/j.jaad.2009.06.046

[10] Pavlović MD Karadaglić DM Kandolf LO Persistent erythema multiforme: a report of three cases. J Eur Acad Dermatol Venereol 2001;15:54–8. 11451326 10.1046/j.1468-3083.2001.00185.x

[11] Sokumbi O Wetter DA Clinical features, diagnosis, and treatment of erythema multiforme: a review for the practicing dermatologist. Int J Dermatol 2012;51:889–902. 22788803 10.1111/j.1365-4632.2011.05348.x

[12] Xu L Zhu Y Yu J More, nursing care of a boy seriously infected with Steven–Johnson syndrome after treatment with azithromycin: a case report and literature review. Medicine 2018;97:e9112. 29505509 10.1097/MD.0000000000009112 PMC5943129

[13] French LE Prins C Erythema multiforme, Stevens-Johnson syndrome, and toxic epidermal necrolysis. In: Dermatology: 2-Volume Set, 3rd Edition, Expert Consult Premium Edition - Enhanced Online Features and Print, Bolognia JL, Jorizzo JL, Rapini RP (eds), Elsevier Limited, 2012, p. 2776.

[14] Hughey LC Approach to the hospitalized patient with targetoid lesions. Dermatol Ther 2011;24:196–206. 21410609 10.1111/j.1529-8019.2011.01395.x

[15] Routt E Levitt J Famciclovir for recurrent herpes-associated erythema multiforme: a series of three cases. J Am Acad Dermatol 2014;71:e146–7. 25219739 10.1016/j.jaad.2014.05.029

[16] Davis MD Rogers RS 3rd Pittelkow MR Recurrent erythema multiforme/Stevens-Johnson syndrome: response to mycophenolate mofetil. Arch Dermatol 2002;138:1547–50. 12472339 10.1001/archderm.138.12.1547

[17] Bakis S Zagarella S Intermittent oral cyclosporin for recurrent herpes simplex-associated erythema multiforme. Australas J Dermatol 2005;46:18–20. 15670172 10.1111/j.1440-0960.2004.00130.x

[18] Chen CW Tsai TF Chen YF Persistent erythema multiforme treated with thalidomide. Am J Clin Dermatol 2008;9:123–7. 18284267 10.2165/00128071-200809020-00006

